# Study on New Dental Materials Containing Quinoxaline-Based Photoinitiators in Terms of Exothermicity of the Photopolymerization Process

**DOI:** 10.3390/ijms24032752

**Published:** 2023-02-01

**Authors:** Ilona Pyszka, Łukasz Skowroński, Beata Jędrzejewska

**Affiliations:** 1 Faculty of Chemical Technology and Engineering, Bydgoszcz University of Science and Technology, 85-326 Bydgoszcz, Poland; 2 Institute of Mathematics and Physics, Bydgoszcz University of Science and Technology, Al. Prof. S. Kaliskiego St. 7, 85-796 Bydgoszcz, Poland

**Keywords:** photopolymerization, dental fillings, dye photoinitiators, photocurable composition

## Abstract

Modern dentistry places great demands on the dental composites used for filling tooth cavities or treating cavitated tooth decay. The aim of the work was to modify the properties of composites by changing the initiators and co-initiators. This was achieved by using initiators based on a quinoxaline skeleton and co-initiators that are derivatives of acetic acid, which is an advantage of these photoinitiating systems due to the elimination of aromatic amines from the photocurable composition. The composites also differed in dental fillers. The effect of the compounds on the exothermicity of the photopolymerization process, the surface morphology of the obtained materials and the maximum compressive strength were determined. The photoinitiating capacity of the two-component systems was tested by the microcalorimetric method using the multifunctional monomer TMPTA, typical for dental filler compositions. The new photoinitiating systems show particularly good efficiency of free radical polymerization initiation, which occurs by the photoinduced intermolecular electron transfer (PET) mechanism. The comparison of the tested systems with camphorquinone, a photoinitiator traditionally used in dentistry, made it possible to observe a decrease in temperature during photopolymerization without a significant decrease in the polymerization rate or increase in photocuring time, as well as a better homogeneity of the surface of the obtained polymeric materials. This indicates that dye–acetic acid derivative systems may be useful in dental applications.

## 1. Introduction

Dental fillings are a large group of materials that differ in both chemical structure and properties. The target place of their application is the human body, which makes the requirements for this group of materials extremely restrictive [1]. The most important is biocompatibility. In addition, the material must not pose a threat to a living organism by, for example, the formation of inflammatory reactions [2]. On the other hand, good mechanical properties of materials for reconstruction allow for high fracture toughness, abrasion, bending, compression and obtaining a degree of hardness similar to the replaced hard tissues of the teeth [3,4,5,6]. An important parameter is also temperature stability, which protects the system against thermal decomposition.

Moreover, the high temperatures during the curing of a dental filling can cause irreversible damage to the pulp [7]. Since the environment of the oral cavity is specific (in addition to physiological fluids such as saliva, it also contains fluids supplied with food), it may contribute to the secretion of potentially harmful chemical compounds from dental fillings into the human body [8]. In addition, dental restorative materials should resemble the tissues they replace, especially for anterior restorations (color, transparency and refractive index) [9]. It is also desirable that they exhibit cariostatic and antibacterial properties.

The above-mentioned requirements are only a part of the most important criteria for the design of dental fillings. Initially, silicon and silica-phosphorus cements were used as plastic restorative materials, which provided good aesthetic results. An alternative to them were acrylic-based fast polymerizing resins, which have been used since 1944. However, it soon turned out that these materials did not work in clinical practice. They showed high polymerization shrinkage leading to the lack of tightness of the fillings, high toxicity in relation to the tooth pulp and rapid color change. In the early 1950s, acrylic resins were improved by adding various inorganic compounds to improve their mechanical properties, especially to reduce polymerization shrinkage. At that time, the first composite materials were developed, which found clinical applications. The discovery by Buonocore in 1955 of the phenomenon of increasing the adhesion of the acrylic resin to acid-etched enamel ushered in the era of “adhesive” dentistry and contributed to the progress in the technology of restorative materials [10].

Considering the chemical composition, dental restoration materials are divided into metals and their alloys, ceramics and polymeric materials. Polymeric materials for the reconstruction of hard dental tissues are a group of modern materials, which include glass ionomer cements, compomers and composites based on resins [7]. 

Composite fillings can harden by either a chemical setting reaction through polymerization between two pastes or a light shone onto it. Chemically cured fillings do not allow you to choose the right shade for patient’s teeth. As a result, the seal may not look aesthetically pleasing. Moreover, the easy formation of micro-fissures, from which it is difficult to remove bacteria, favors the development of caries. Most modern composite resins are light-cured photopolymers, meaning that they harden with light exposure. Their quality is very good, and they come in shades that allow you to choose the right color for each patient [11,12,13]. 

Standard dental polymer compositions consist of two main components: a liquid organic matrix undergoing cross-linking polymerization and an inorganic powder filler that significantly impacts the composite’s final properties. A liquid organic matrix is a monomer composite containing: multifunctional monomers, photoinitiators and co-initiators [12,13,14,15,16,17,18]. Reinforcing fillers are, for example, quartz, silicate glass, etc., inhibitors that prevent premature polymerization during storage; photostabilizers stabilizing the color of the filling and compounds that allow you to match the color of the filling to the natural color of the patient’s teeth [13,14,15,18,19]. 

Initiation of photopolymerization [13,16] requires the use of photoinitiators, i.e., compounds that generate free radicals after photon absorption [20]. Their content in the composition is up to 1% [13]. The most commonly used photoinitiator for dental polymeric materials is camphorquinone, which is sensitive to blue light (460–480 nm) [13,16,18,20]. Its reactivity is increased in the presence of co-initiators acting as electron donors [13,16,20]. The typical co-initiators are tertiary amines, e.g., 4-*N,N*-dimethylamino-phenylethanol and 4-dimethylaminoethyl benzoate [13,18,21]. 

In addition to camphorquinone, a large group of chemical compounds is described in the literature as photoinitiators of free radical polymerization [22,23]. Generally, they can be divided into Norish I and II initiators. The latter are usually two-component systems containing a light absorber and a co-initiator (electron donor) [24]. Type I photoinitiators are usually aromatic carbonyl compounds containing additional functional groups to facilitate homolytic photodissociation. Depending on the type and location of the functional group, the bond cleavage occurs in the α position (α-photodissociating initiators) [25,26] or in the β-position (β-photodissociating initiators) in relation to the carbonyl group [27]. The I group of photoinitiators also includes organic compounds containing weak bonds such as O-O, S-S, N-S and C-N [25,26,27].

Type II photoinitiators, called photoinitiating systems, generate radicals in bimolecular processes. They consist of a chromophore and a suitable co-initiator to form photo-redox pairs. Typical type II photoinitiators are benzophenone and its derivatives, thioxanthones, benzil, quinones, and organic dyes, while a wide group of carboxylic acids, tertiary amines, borate salts and others are applied as the electron donors [25,28,29,30,31,32,33]. 

Modern dentistry places great demands on dental filling materials composites. Modifications of the composite properties can be achieved by changing the composition of the polymer matrix. Therefore, our research aimed to improve the properties of polymeric materials by using new initiators and co-initiators in the photocurable composition. New organic compounds based on quinoxaline skeleton were used as initiators. Aromatic amines traditionally used as co-initiators have been replaced with acetic acid derivatives. 

Since the distribution of the filler in the polymer matrix affects the surface quality through the formation of fractures and other mechanical damage that can cause undesirable biological interactions and adhesion of the bacterial plaque to the reconstruction material, an additional aim of these studies was to determine the homogeneity of the distribution of three commercial fillers in the organic matrix. Thus, the effect of these compounds on the exothermicity of the photopolymerization process, surface morphology and hardness of the obtained polymeric materials was investigated.

## 2. Results and Discussion

Currently, many research groups seek multifunctional and versatile organic and inorganic molecules that can be adapted in dentistry as new, useful materials for the oral cavity [12,16,34]. However, the road from discovering a suitable material to designing a tangible product is long. Composite materials for dental fillings need to be biocompatible materials with optimal physical, mechanical, chemical and aesthetic properties. Engineers are constantly trying to improve current dental materials to minimize their drawbacks and create an elite product that is more durable and less toxic. The emphasis is mainly on modifying the chemical composition of polymeric materials, both within their matrix and filler [12,13,14,15,16,17,18]. For example, through a properly selected filler, it is possible to reduce the temperature below the threshold corresponding to irreversible damage to the pulp or to obtain high durability, strength and very good adhesion [35,36,37]. The studies described here are limited to composite materials consisting of novel visible light photoinitiators synthesized based on the quinoxaline backbone, acetic acid derivatives being a co-initiator and three different commercial fillers.

### 2.1. Exothermicity of the Polymerization Process

The type II photoinitiators consist of a suitable dye as a primary absorber and a co-initiator [24]. In the present work, four quinoxaline-based compounds were synthesized [32,38] and used as new sensitizers because they have a rigid structure that prevents rotation of the benzene rings or isomerization, allowing the elimination of excited state deactivation channels. Moreover, their synthesis is simple and inexpensive. They have appropriate spectroscopic properties as well [32,38]. The compounds absorb ultraviolet and visible light, allowing dental lamps to be used as light sources for photoinitiation of the polymerization process. A desirable feature is also the ability to form a long-lived excited state, which enables the photopolymerization reaction to proceed by the intermolecular electron transfer (PET) mechanism [28,39,40].

**Figure 1:**
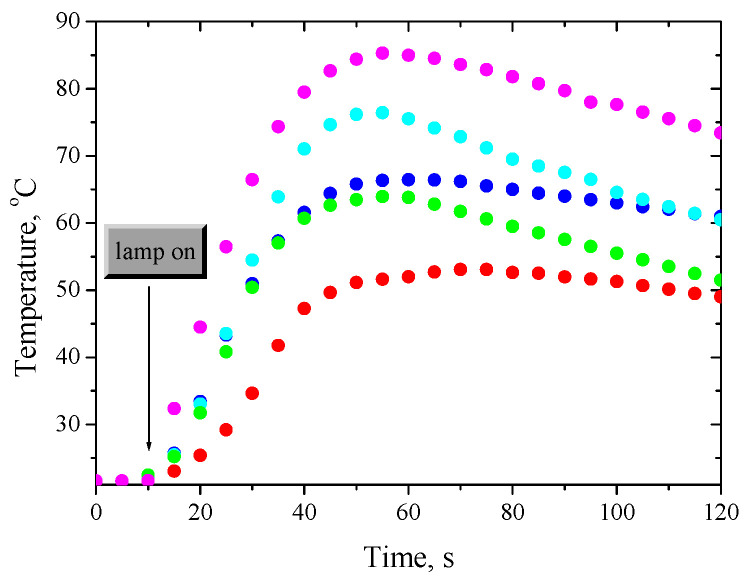
Comparison of temperature changes during irradiation of monomer composites containing synthesized photoinitiators and commercial camphorquinone. Photoinitiating systems include: 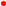
—Ph1 (dibenzo[a,c]phenazine), 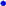
—Ph2 (benzo[a]phenazine), 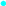
—Ph3 (11*H*-indeno[1,2-b]quinoxalin-11-one), 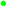
—Ph4 (6*H*-indolo[2,3-b]quinoxaline) and 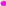
—CQ (camphorquinone) as photoinitiator, and 1-naphthoxyacetic acid (NAA; c = 0.1 M) as co-initiator. I = 15 mW/cm^2^.

Figure 1 and Figure S1 show the kinetic curves recorded for the tested photoinitiating systems during the free radical polymerization of TMPTA.

The initial rate of free radical polymerization of TMPTA was determined from the inclination of the rectilinear fragment of the kinetic curves (Figure 1). The data, along with the maximum temperature rise during photopolymerization, are summarized in Table S1.

**Figure 2:**
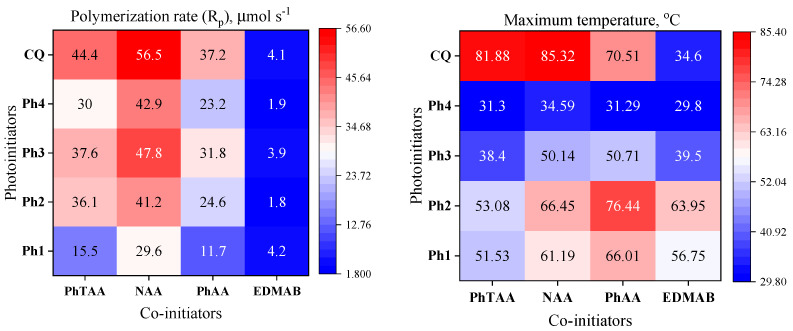
Heatmaps of the polymerization rate (μmol s^−1^) (**left**) and maximum temperature rise (**right**) during photoinitiated polymerization of TMPTA; photoinitiating systems consisted of photoinitiators: dibenzo[a,c]phenazine (Ph1), benzo[a]phenazine (Ph2), 11*H*-indeno[1,2-b]quinoxalin-11-one (Ph3), 6*H*-indolo[2,3-b]quinoxaline (Ph4) and camphorquinone (CQ), and co-initiators: (phenylthio)acetic acid (PhTAA), 1-naphthoxyacetic acid (NAA), phenoxyacetic acid (PhAA) and ethyl 4-dimethylaminobenzoate (EDMAB).

To verify the photoinitiating abilities of the tested systems, the initial rates of photoinitiation of radical polymerization by the applied photoinitiators (Ph1–Ph4) were comparable to the rates obtained for polymerization initiated by a commercial CQ photoinitiator for dentistry (Figure 2). To maintain the same experimental conditions (number of absorbed photons), the concentration of CQ in the composition was 440–1430 times higher than the tested photoinitiators due to the lower molar absorption coefficient in the visible range (e.g., CQ 8 M^−1^ cm^−1^ vs. Ph1 11,000 M^−1^ cm^−1^ at 400 nm). This made it possible to obtain thick polymer layers (3 mm) that did not contain significant amounts (approx. 0.675 M) of unreacted CQ and its decomposition products. The initial polymerization rates of TMPTA are comparable, and a hard polymer glaze is obtained after about 30 s of irradiation (Table S1). In addition, the temperature of the photopolymerization process initiated by systems based on photoinitiators (Ph1–Ph4) and acetic acid is lower than for a CQ (Table 1), which allows for the safe use of these systems in dentistry.

The tested systems are effective visible light photoinitiators in dental compositions. Their ability to initiate a chain reaction depends on both the type of light absorber (dyes) and the co-initiator (acids). Differences in their structure affect the properties of acids, e.g., oxidation potential, reactivity and the type of radicals formed due to the PET process. These, in turn, are crucial for initiating the polymerization reaction [32,38,41,42]. The choice of co-initiators resulted from the need to eliminate amines from the photocurable composition. For comparative purposes, ethyl 4-dimethylaminobenzoate (EDMAB), a commercial dental initiator, was used. Analyzing the results, it was justified to replace aromatic amines with acetic acid derivatives in dental compositions. Compositions containing NAA and PhTAA showed higher polymerization rates than the commonly used amine EDMAB. In practice, this means shortening the polymerization time necessary to obtain a hard polymer. In addition, the replacement of the oxygen atom by sulfur in the co-initiator structure (PhAA vs. PhTAA) increases the rates of TMPTA photopolymerization. This is associated with a more efficient PET process (lower ∆G_ET_ [43,44]) and the different reactivity of the radicals obtained in the secondary reactions that follow the electron transfer process. The polymerization mechanism and the composition of the post-reaction mixture determined based on the ^1^H NMR technique were described in our previous paper [45].

In practice, the photopolymerization process is conducted directly in the patient’s mouth. During photocuring, the temperature increases due to the exothermic nature of the polymerization process, as well as the heat emitted by the dental lamp [46,47,48,49,50]. The increase in temperature during photopolymerization is of great importance because it affects not only the composite properties [51] but, above all, the pulp—the tissue that builds the tooth being filled [52]. Literature reports show that an increase in pulp temperature by 5.5 °C in relation to the initial temperature causes irreversible inflammation of the pulp, which covers as much as 15% of its volume, while an increase in temperature by 11.2 °C causes as much as 60% of the pulp to undergo irreversible changes [53].

Light sources with high energy efficiency cause a greater increase in temperature [48]. Polymerization halogen lamps have a greater thermal effect than argon lasers or LED lamps [46,54]. The thermal effect also increases with the length of exposure time [46].

**Figure 3:**
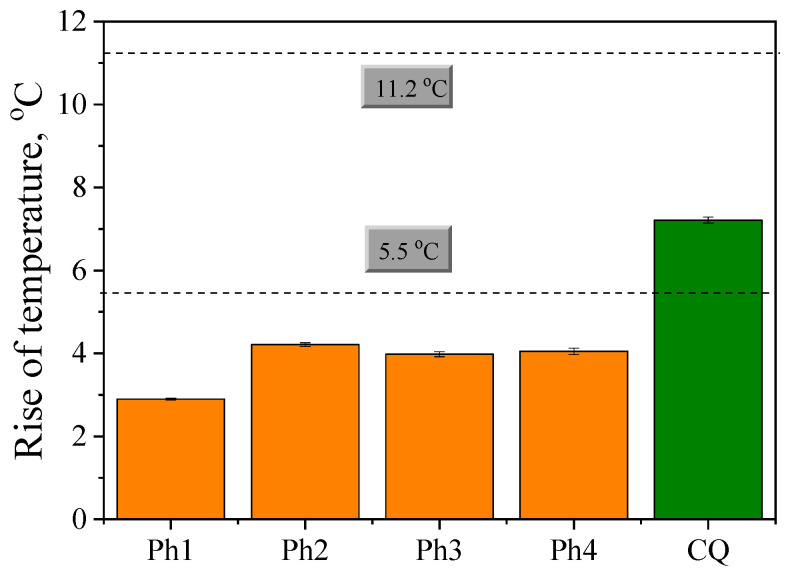
The mean values with a standard deviation of the maximum temperature increase during irradiation of the tested polymeric materials. The photoinitiating systems contain different light absorbers marked in Figure and the same co-initiator (phenylthio)acetic acid (PhTAA) and the dental filler DF3 (i-FIX LC Light Curing Glass Ionomer Filling Cement). The light intensity of the dental lamp was 15 mW/cm^2^.

The observed increase in temperature during the photopolymerization is of the order of 40–60 °C (Figure 2, Table S1). The addition of a commercial filler to the monomer composite significantly reduces the temperature (Figure S2, Tables S2–S4). Figure 3 and Figure S3 illustrate the maximum values of the temperature increases for the tested polymeric materials determined based on temperature measurements during the photopolymerization process.

The dashed lines in Figure 3 and Figure S3 indicate the two pulp temperature tolerance thresholds. The first limit represents a temperature increase of 5.5 °C [53,55,56]. This value is not exceeded when the polymerization is initiated by synthesized Ph1–Ph4 photoinitiators. It should be emphasized that for the commercial CQ photoinitiator, the maximum temperature difference achieved during irradiation of the composition is higher than 5.5 °C but does not exceed the upper-temperature tolerance threshold, i.e., 11.2 °C. Taking into account the values of the maximum temperature difference obtained for CQ, i.e., 6.2 °C, it can be concluded that the tested photoinitiators (Ph1–Ph4) in composition with commercial dental fillers DF1, DF2 and DF3, respectively, do not exceed the temperature tolerance threshold of the pulp.

### 2.2. Surface Morphology

**Figure 4:**
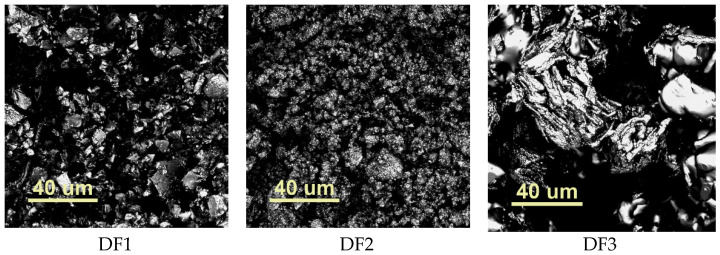
Images taken with a confocal optical microscope (COM). The scanned area was 129 µm × 129 µm (for the ×100 lens); abbrev: DF1—filler Ketac Fil Plus, DF2—Kromoglass 2 and DF3—i-FIX LC Light Curing Glass Ionomer Filling Cement.

Determining morphology enables a complete characterization of materials with potential dental applications. It facilitates the assessment of the homogeneity of the filler distribution in the organic matrix, which affects the exploitation of the obtained filling. The surface imaging of the tested sample was performed using a confocal optical microscope (COM) Lext OLS4000 from Olympus. Figure 4 shows the microscopic characteristics of the fillers used.

The fillers used were DF1 and DF2, which contains crushed fluoroaluminosilicate glass, quartz and glass fiber. On the other hand, the DF3 filler is a typical glass fiber obtained from a water glass made of sodium and potassium silicates [57]. It is very often used as composite reinforcement because it is characterized by high durability, strength and very good adhesion [58,59,60].

Manufacturers of commercial materials DF1, DF2 and DF3 do not provide the average size of the filler particles; however, the analysis of the obtained images shows that the glass particles contained in these materials are of the order of micrometers.

**Figure 5:**
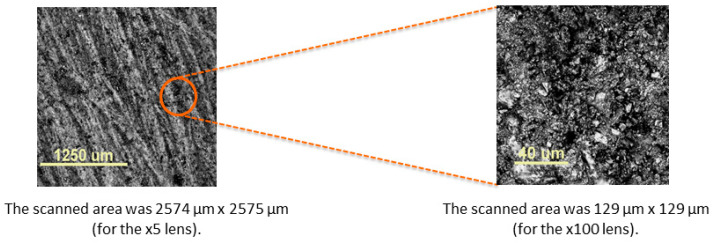
Images taken with a confocal optical microscope (COM). The scanned area was 2574 µm × 2575 µm (for the ×5 lens) and 129 µm × 129 µm (for the ×100 lens); abbrev.: dibenzo[a,c]phenazine (Ph1), (phenylthio)acetic acid (PhTAA), DF1—filler Ketac Fil Plus.

Figure 5 presents images of the surface of the obtained material containing: Ph1, PhTAA and DF1 obtained at 5× magnification (the scanned area was 2574 μm × 2574 μm) and at 100× magnification (the scanned area was 129 μm × 129 μm).

**Figure 6:**
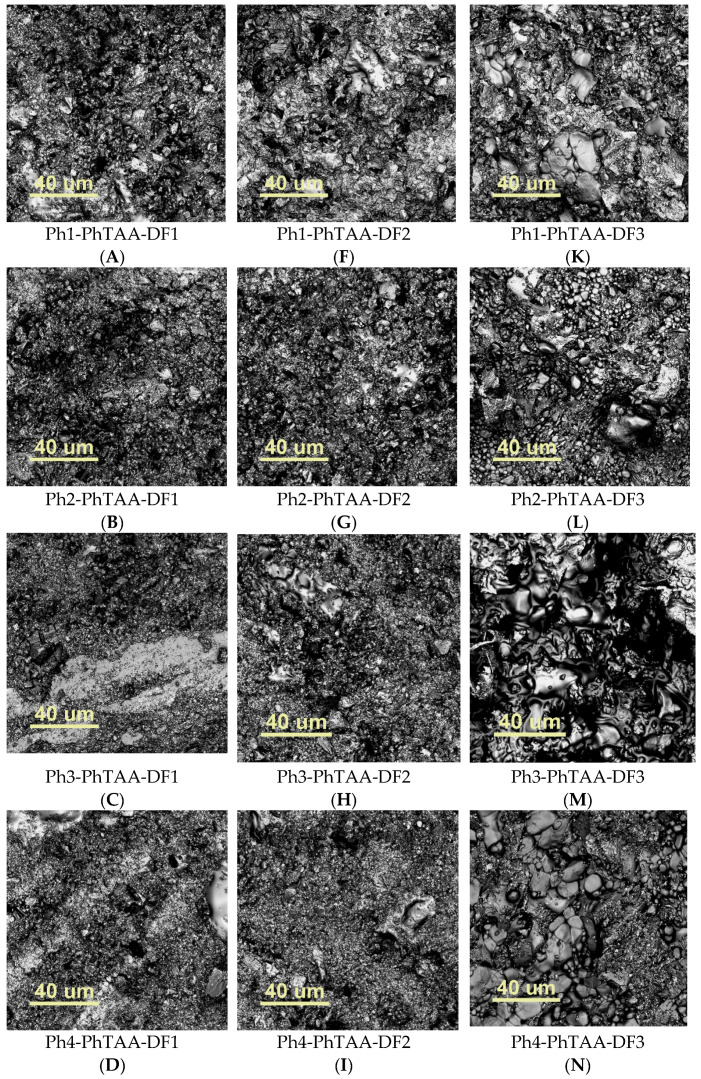

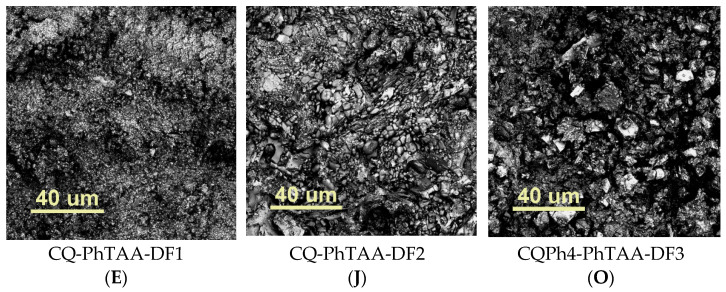
Images taken with a confocal optical microscope (COM); abbrev.: dibenzo[a,c]phenazine (Ph1), benzo[a]phenazine (Ph2), 11*H*-indeno[1,2-b]quinoxalin-11-one (Ph3), 6*H*-indolo[2,3-b]quinoxaline (Ph4), (phenylthio)acetic acid (PhTAA), DF1—filler Ketac Fil Plus, DF2—Kromoglass 2 and DF3—i-FIX LC Light Curing Glass Ionomer Filling Cement.

Figure 6 shows exemplary photos of the surface of the tested materials illustrating significant morphological differences in the top layer of the analyzed samples.

The materials containing the DF1 (A–E) filler are characterized by a smaller particle size with a significant regularity of shapes in comparison to the DF2 (F–J) and DF3 (K–O) materials. Some irregularity characterizes the surface of these composites. This is a typical feature of composites with a filler particle size of several μm [16]. Despite visible agglomeration areas, their distribution seems relatively homogeneous. Composites containing DF2 (F–J) and DF3 (K–O) show a greater tendency to form filler clusters.

### 2.3. Compressive Strength

Compressive strength (R) is the mechanical property of dental restorative materials that determine their hardness. The lack of standards defining limit values for dental materials justifies comparing the R-values of experimental materials with the values determined for commercial dental materials.

**Figure 7:**
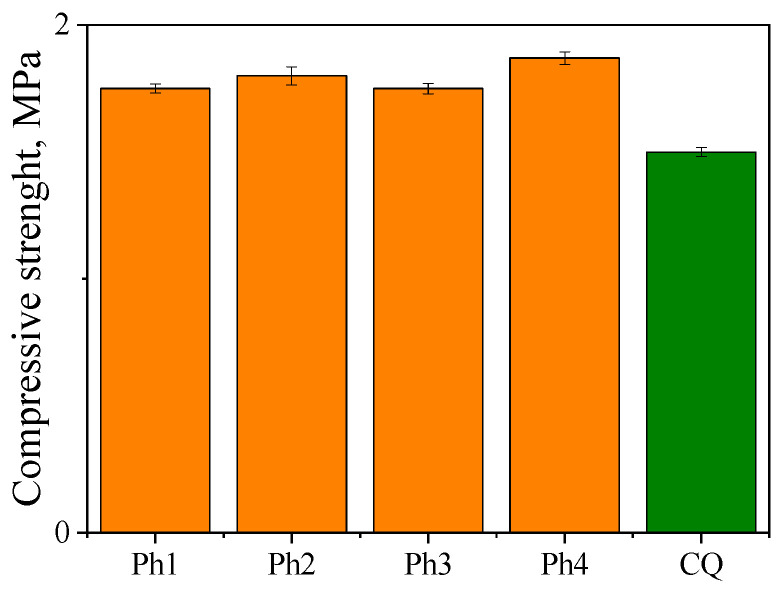
Dependence of the compressive strength on the type of photoinitiator: dibenzo[a,c]phenazine (Ph1), benzo[a]phenazine (Ph2), 11*H*-indeno[1,2-b]quinoxalin-11-one (Ph3), 6*H*-indolo[2,3-b]quinoxaline (Ph4) and camphorquinone (CQ); co-initiator: (phenylthio)acetic acid (PhTAA); DF1–filler Ketac Fil Plus. The plot shows the mean values with standard deviation.

Figure 7 shows the maximum compressive strength at which samples of the tested materials containing filler DF1 are broken under compression.

The composition of a material has the greatest influence on compressive strength. In particular, it is the composition of the organic matrix, the type of filler and its content, and the curing method. Compressive strength tests refer to the real situation in the patient’s mouth when chewing food. Photoinitiators we synthesized (Ph1–Ph4) used in photocuring mixtures together with glass ionomer DF1 allow us to obtain exceptionally hard dental fillings. All the polymeric materials have better compressive strength (Table S5) than the dental filling obtained based on a commercial photoinitiator—camphorquinone. They show compressive strength of 1.20–1.90 MPa. Based on compressive strength, which is the most commonly defined mechanical property of dental filling materials, it can be assumed that the designed systems may find potential application in dental composites.

## 3. Materials and Methods

### 3.1. Materials

**Figure 8:**
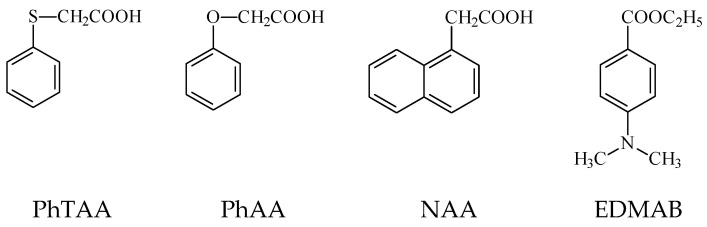
Co-initiator structures used in the photopolymerizable compositions: (phenylthio)acetic acid (PhTAA), 1-naphthoxyacetic acid (NAA), phenoxyacetic acid (PhAA), and ethyl 4-dimethylaminobenzoate (EDMAB).

Camphorquinone (CQ)—commercial photoinitiator, co-initiators: (phenylthio)acetic acid (PhTAA), 1-naphthoxyacetic acid (NAA), phenoxyacetic acid (PhAA), ethyl 4-dimethylaminobenzoate (EDMAB) and trimethylolpropane triacrylate (TMPTA) monomer were purchased from Sigma-Aldrich. The co-initiator structures are shown in Figure 8.

**Figure 9:**
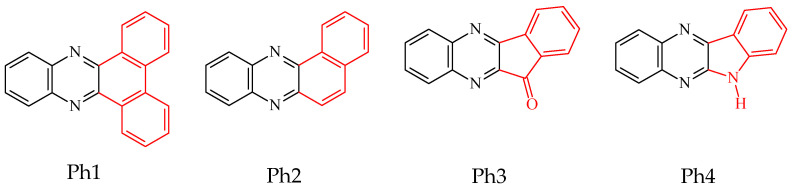
Structures of photoinitiators used in the photopolymerizing composition; dibenzo[a,c]phenazine (Ph1), benzo[a]phenazine (Ph2), 11*H*-indeno[1,2-b]quinoxalin-11-one (Ph3), and 6*H*-indolo[2,3-b]quinoxaline (Ph4).

Four organic compounds with different molecular structures were selected as photoinitiators of the polymerization reaction conducted in the presence of various dental fillers, i.e., Ph1—dibenzo[a,c]phenazine, Ph2—benzo[a]phenazine, Ph3—11*H*-indeno[1,2-b]quinoxalin-11-one, and Ph4—6*H*-indolo[2,3-b]quinoxaline. Their structures are shown in Figure 9, and their synthesis is described in [32,34].

Photopolymerization was conducted in the presence of three types of dental fillers, i.e., DF1-filler Ketac Fil Plus purchased from 3M-ESPE Co, DF2 Kromoglass 2 from Lascod and DF3 i-FIX LC Light Curing Glass Ionomer Filling Cement from Vita Zahnfabrik H.Rauter GmbH & Co.KG, Bad Sackingen, Germany.

The tested polymer composite materials for dental fillings consisted of a monomer composite and a filler. The monomer composite was a mixture of 0.9 g of TMPTA, 0.1 g of 1-methyl-2-pyrrolidone, a photoinitiator and a co-initiator. The concentration of photoinitiators ranged from 1.05 × 10^−3^ M to 4.73 × 10^−4^ M, depending on the molar absorption coefficient (Table 1) [32,34]. The concentration of the co-initiators was 0.1 M. The filler was a reinforcing powder—commercial material containing, among others, quartz, silicate glass and fiberglass (composition depending on the manufacturer). The ratio of monomer composite to filler was 3:7 wt.%. The appropriate amount of filler was weighed and mixed with the prepared photocurable composition in a mortar.

### 3.2. Methods

#### 3.2.1. Photopolymerization

**Figure 10:**
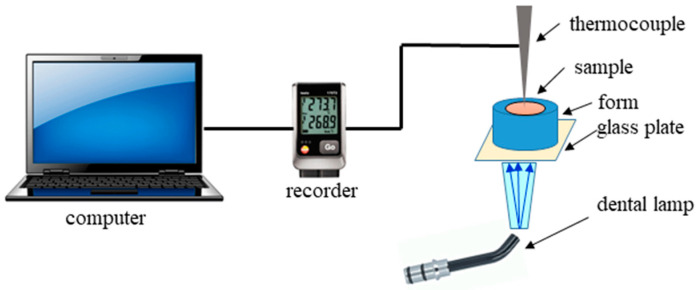
Schematic diagram of the system used to measure temperature changes during the photopolymerization process for the tested composite materials.

The microcalorimetric method investigated the kinetics of photoinitiated polymerization [32,61]. Changes in the heat released during the polymerization process were recorded using the system shown in Figure 10.

The photocurable composition was placed in a Teflon ring with a diameter of 10 mm and a thickness of 3 mm, protected on one side with a glass plate. The sample was irradiated with blue light from a dental halogen lamp—Cromalux 75, MEGA-PHYSIK GMBH & CO, Rastatt, Germany through the bottom window. A threshold time was needed to stabilize the temperature in a sample before irradiation was 10 sec. The delay time was the same for all samples. The distance of the optical fiber from the surface of the glass plate was 0.5 cm. A thermocouple (stainless steel, type K, Φ 4 mm, RTD Thermometer HD 2107.1, Delta OHM, Padova, Italy) measured temperature changes during polymerization. It was inserted into the polymerizing composition from the top so that its tip was in contact with the surface of the glass plate. The intensity of the light (measured with the Field Master meter, Coherent, Santa Clara, California, USA) was 15 mW/cm^2^. The distance of the sample from the light source was the same for all measurements. The temperature was recorded every 5 s for 2 min. Connecting the thermocouple to the recorder (Delta OHM HD 40.1, Padova, Italy) enabled temperature measurement with an accuracy of ±0.1 °C. Three measurements were made for each of the tested samples.

The commercial photoinitiator camphorquinone (CQ) was used to compare the effectiveness of initiating the polymerization reaction by the tested systems. Its concentration was 0.675 M.

#### 3.2.2. Surface Morphology

The morphology of the samples was investigated using the confocal optical microscope (COM) Lext OLS4000 from Olympus, Tokyo, Japan. The scanned area was 2574 µm × 2575 µm (for the ×5 lens) and 129 µm × 129 µm (for the ×100 lens). The monomer composite for the preparation of the samples for surface morphology studies contained the synthesized photoinitiators (Ph1–Ph4), the PhTAA co-initiator and commercially available fillers DF1, DF2 and DF3, respectively. The surface morphology studies were aimed at evaluating the effect of commercial dental fillers on the homogeneity of the organic matrix. It is well known [35,36,37] that the shape, size and tendency to aggregate the filler affects the subsequent use of the dental filling since it is responsible for the formation of fractures and other mechanical damage.

#### 3.2.3. Compressive Strength

Compressive strength tests were carried out using Instron universal testing systems model 6800 (Norwood, MA, USA). The following parameters were set: test speed—1 mm min^−1^, initial force 10 N, test end—the moment of sample destruction. Samples obtained from monomer composites containing photoinitiators: Ph1, Ph2, Ph3, Ph4 and CQ, co-initiator PhTAA and filler DF1 were selected for the study. The measurement for each of the tested materials was repeated five times. The samples of the tested materials had the shape of a cuboid with dimensions of 5 × 10 × 10 mm. The sample was in the middle of the lower clamping table, which was parallel to the upper table. The compressive force acted perpendicularly to both tables along a dimension of 5 mm. A probe attached to the load cell on the upper table was moved onto the specimen at a low rate of travel (1 mm min^−1^), compressing the material until it failed.

Compressive strength was calculated using Formula (1) [62]:

(1) $$R=\frac{F}{A}\;\;\left(\mathrm{MPa}\right)$$

where *F* is the greatest applied load recorded during compression of the sample in N, and *A* is the area of the initial cross-section of the sample in mm^2^.

## 4. Conclusions

Four organic compounds containing a quinoxaline skeleton have been evaluated as potentially valuable visible light photoinitiators for dentistry. These dyes, in combination with acetic acid derivatives (PTAA, PAA and NAA), show a particularly good ability to photoinitiate free radical polymerization of TMPTA. The effectiveness of photoinitiating systems is comparable to camphorquinone, a commercial photoinitiator used in dentistry. In addition, tests conducted in the presence of commercial dental fillers (DF1, DF2, DF3) showed that for any photoinitiator, the increase in temperature during the polymerization of TMPTA did not exceed the upper threshold of the temperature tolerance for the tooth pulp. Furthermore, the obtained dental fillings show surface homogeneity, with the particle size of the fillers being several μm. The fillings containing the DF1 filler also have better compressive strength than the dental filling obtained based on commercial photoinitiator—camphorquinone. This suggests that the investigated two-component photoinitiating systems may have potential applications in dentistry. However, the studies described here concern only a preliminary assessment of the usefulness of the synthesized compounds as photoinitiators and focus on the design of photoinitiating systems that effectively initiate radical polymerization in a short time at a low temperature increase. The practical application of photocurable compositions in dentistry needs more advanced research. Further studies will include, among others (i) testing the depth of hardening, (ii) study of the compounds released in the oral cavity during the use of the dental materials, (iii) bioassays, (iv) determination of toxicity, and (v) the effect of the commercially available polishing systems on their surface topography and roughness.

## Figures and Tables

**Table 1:** Photoinitiator concentration in the tested polymer composite: dibenzo[a,c]phenazine (Ph1), benzo[a]phenazine (Ph2), 11*H*-indeno[1,2-b]quinoxalin-11-one (Ph3), 6*H*-indolo[2,3-b]quinoxaline (Ph4) and camphorquinone (CQ).

Photoinitiator	Ph1	Ph2	Ph3	Ph4	CQ
Concentration (M)	4.75 × 10^−4^	1.10 × 10^−4^	1.55 × 10^−3^	1.05 × 10^−3^	0.675

## Data Availability

The data is not publicly available apart from the data contained in the article or Supplementary Materials.

## Supplementary Materials

The supporting information can be downloaded at: https://www.mdpi.com/article/10.3390/ijms24032752/s1.
