# Supplementary material for: Study on New Dental Materials Containing Quinoxaline-Based Photoinitiators in Terms of Exothermicity of the Photopolymerization Process
Source: Int J Mol Sci. 2023 Feb 1;24(3):2752. doi: 10.3390/ijms24032752 (PMC9917465; doi:10.3390/ijms24032752)
Supplement: Supplementary file 1 [file ijms-24-02752-s001.zip › ijms-2097049-supplementary.pdf]

Supporting information

Study on new dental materials containing quinoxaline-based photoinitiators in terms of exothermicity of the photopolymerization process

Ilona Pyszka [1], Łukasz Skowroński[2], Beata Jędrzejewska[3]

[1] Faculty of Chemical Technology and Engineering, Bydgoszcz University of Science and Technology, 85-326 Bydgoszcz, Poland
[2] Institute of Mathematics and Physics, Bydgoszcz University of Science and Technology, Al. Prof. S. Kaliskiego St. 7, 85-796 Bydgoszcz, Poland
[3] Faculty of Chemical Technology and Engineering, Bydgoszcz University of Science and Technology, 85-326 Bydgoszcz, Poland

* Correspondence: Ilona.Pyszka@pbs.edu.pl; Tel.: +48-52-374-9039

Table of contents **Page**

Temperature changes during irradiation – Figure S1 ………………………………………………………….. S2
Effect of dental filler on temperature during polymerization – Figure S2 …………………………………. S2
The maximum temperature increase during irradiation – Figure S3 ………………………………………. S3
Initial rate of photoinitiated polymerization and maximum temperature rise – Table S1………………… S3
Initial rate of photoinitiated polymerization and maximum temperature rise for composition with filler – Table S2 –S4…………………………………………………………………………………………………… S3-S4
Compressive strength for the tested dental fillings – Table S5 ……………………………………. S4

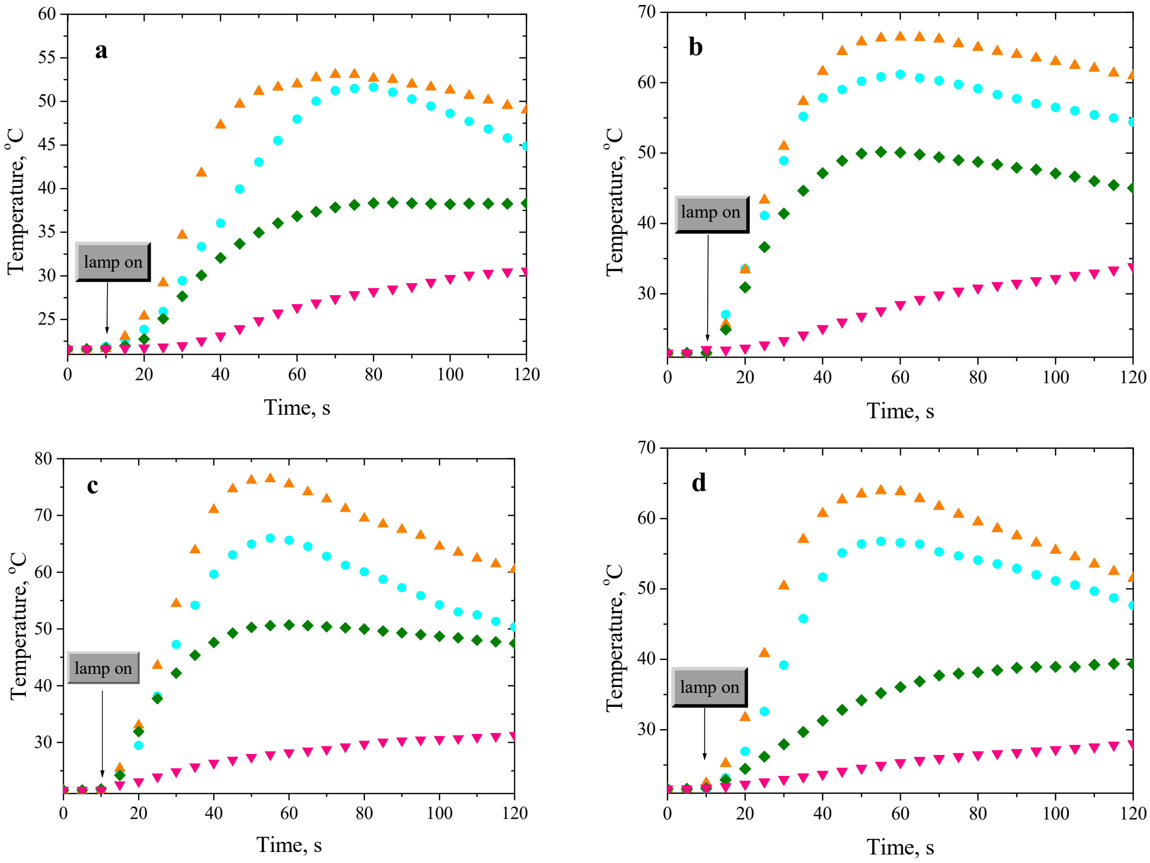

Figure S1. Temperature changes during irradiation of the tested monomer composites. Initiating system: photoinitiator: **a**-Ph1, **b**-Ph2, **c**-Ph3, **d**-Ph4; co-initiator: ▲-NAA, ●-PhTAA, ◆-EDMAB, ▼-PAA (c = 0.1 M). I =15 mW/cm^2. Trimethylolpropane triacrylate (TMPTA) was used as monomer. Abbrev.: dibenzo[a,c]phenazine (Ph1), benzo[a]phenazine (Ph2), 11*H*-indeno[1,2-b]quinoxalin-11-one (Ph3), 6*H*-indolo[2,3-b]quinoxaline (Ph4), camphorquinone (CQ), (phenylthio)acetic acid (PhTAA), 1-naphthoxyacetic acid (NAA), phenoxyacetic acid (PhAA) and ethyl 4-dimethylaminobenzoate (EDMAB) as co-initiators

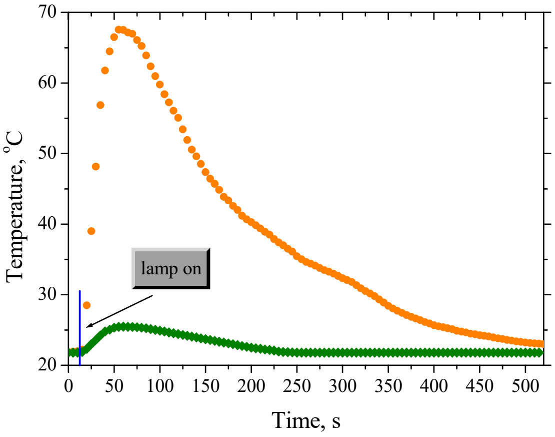

Figure S2. Kinetic curves showing the effect of dental filler on temperature during polymerization initiated by Ph2 in the presence of 1-naphthoxyacetic acid (NAA; c = 0.1 M). ●- lack of filler, ◆- presence of filler (DF1). Trimethylolpropane triacrylate (TMPTA) was used as monomer. The light intensity of the dental lamp was 15 mW/cm^2.

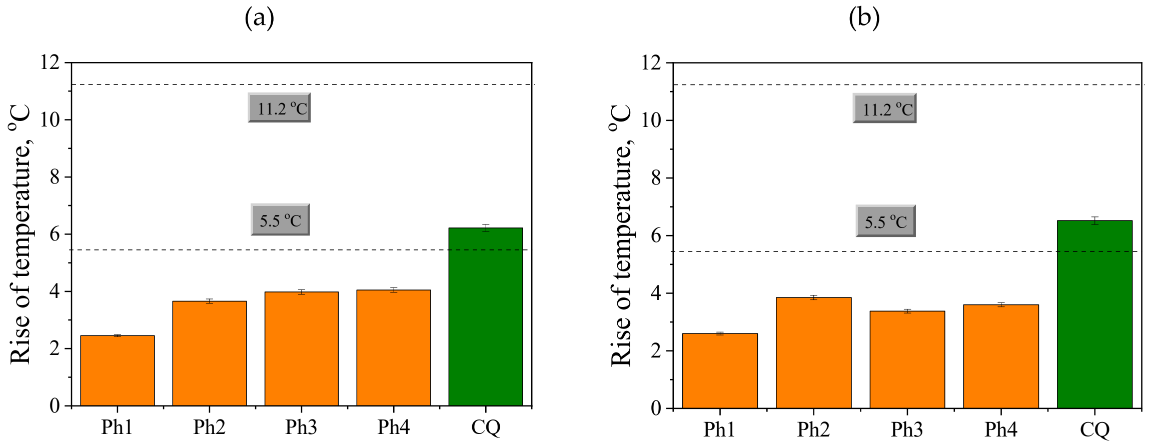

Figure S3. The mean values with standard deviation of the maximum temperature increase during irradiation of the tested polymeric materials. The photoinitiating systems contain different light absorbers (photoinitiators) marked in Figure (Ph1-4 and CQ) and the same co-initiator PhTAA. As the dental filler DF1 (a) and DF2 (b) were used, respectively. The light intensity of the dental lamp was 15 mW/cm^2.

Table S1. Initial rate of photoinitiated polymerization (R_p) and maximum temperature (T_{max}) rise for the tested composites included dibenzo[a,c]phenazine (Ph1), benzo[a]phenazine (Ph2), 11*H*-indeno[1,2-b]quinoxalin-11-one (Ph3), 6*H*-indolo[2,3-b]quinoxaline (Ph4) and camphorquinone (CQ) as photoinitiators and (phenylthio)acetic acid (PhTAA), 1-naphthoxyacetic acid (NAA), phenoxyacetic acid (PhAA) and ethyl 4-dimethylaminobenzoate (EDMAB) as co-initiators. Trimethylolpropane triacrylate (TMPTA) was used as monomer.

Dye	R_p [μmol s^{-1}]				T_{max} [°C]			
	PhTAA	NAA	PhAA	EDMAB	PhTAA	NAA	PhAA	EDMAB
Ph1	15.5±0.10	29.6±0.18	11.7±0.12	4.2±0.15	51.53±0.94	61.19±0.98	66.01±1.08	56.75±0.95
Ph2	36.1±0.21	41.2±0.21	24.6±0.10	1.8±0,12	53.08±1.00	66.45±1.05	76.44±1.12	63.95±1.10
Ph3	37.6±0.21	47.8±0.10	31.8±0.10	3.9±0,14	38.40±1.02	50.14±1.09	50.71±0.99	39.50±1.15
Ph4	30.0±0.20	42.9±0.12	23.2±0.14	1.9±0,11	31.30±1.10	34.59±1.12	31.29±0.98	29.80±1.20
CQ	44.4±0.10	56.5±0.11	37.2±0.12	4.1±0,10	81.88±0.99	85.32±1.05	70.51±1.00	34.60±1.15

Table S2. Initial rate of photoinitiated polymerization (R_p) and maximum temperature (T_{max}) rise for the tested composites included DF1 filler

Dye	R_p [μmol s^{-1}]				T_{max} [°C]			
	PhTAA	NAA	PhAA	EDMAB	PhTAA	NAA	PhAA	EDMAB
Ph1	1.2±0.10	2.1±0.11	0.9±0.10	4.5±0.11	24.05±1.02	28.32±0.97	23.01±1.12	25.02±1.25
Ph2	2.7±0.11	3.0±0.12	1.9±0.12	2.3±0,11	25.26±1.20	30.05±1.20	24.97±1.18	26.76±1.22
Ph3	2.9±0.10	3.5±0.10	2.5±0.11	4.4±0,12	24.68±1.15	27.89±1.22	23.00±1.20	25.34±1.15
Ph4	2.3±0.10	3.2±0.11	1.8±0.10	2.4±0,10	25.00±0.98	28.88±1.08	24.04±1.28	26.76±0.99
CQ	3.2±0.10	3.1±0.10	2.6±0.10	4.8±0.10	27.82±0.99	32.51±1.05	26.55±1.36	28.22±0.98

Table S3. Initial rate of photoinitiated polymerization (R_p) and maximum temperature (T_{max}) rise for the tested composites included DF2 filler

Dye	R_p [μmol s^{-1}]				T_{max} [°C]			
	PhTAA	NAA	PhAA	EDMAB	PhTAA	NAA	PhAA	EDMAB
Ph1	0.8±0.10	1.6±0.11	0.5±0.10	3.2±0.11	24.20±0.99	28.78±1.25	23.98±1.24	26.92±0.99
Ph2	2.1±0.11	2.4±0.10	1.4±0.11	1.4±0.10	25.45±1.20	31.09±1.28	25.76±1.22	27.88±1.16
Ph3	2.1±0.12	2.8±0.10	2.0±0.12	3.5±0.11	24.98±1.32	29.89±1.16	24.20±1.12	26.31±1.14
Ph4	1.7±0.10	2.1±0.12	1.2±0.10	1.6±0.10	25.20±1.14	30.58±1.14	25.66±1.10	27.54±1.16
CQ	2.5±0.11	2.2±0.13	2.1±0.10	3.8±0.13	28.12±1.16	31.57±1.22	27.49±1.14	29.23±1.24

Table S4. Initial rate of photoinitiated polymerization (R_p) and maximum temperature (T_{max}) rise for the tested composites included DF3 filler

Dye	R_p [µmol s^{-1}]				T_{max} [ºC]			
	PhTAA	NAA	PhAA	EDMAB	PhTAA	NAA	PhAA	EDMAB
**Ph1**	1.0±0.10	1.9±0.10	0.7±0.11	4.2±0.11	24.50±1.05	29.99±1.12	24.51±1.35	27.12±1.12
**Ph2**	2.4±0.11	2.7±0.10	1.6±0.12	1.8±0.14	25.81±0.98	32.45±1.15	25.91±1.25	28.86±1.12
**Ph3**	2.5±0.12	3.1±0.11	2.1±0.11	3.9±0.14	25.58±0.99	29.69±1.16	24.09±1.20	26.38±1.14
**Ph4**	2.0±0.10	2.8±0.10	1.5±0.15	1.9±0.10	25.65±0.98	29.89±1.16	26.09±1.14	27.36±1.21
**CQ**	2.9±0.10	2.7±0.11	2.4±0.14	4.1±0.11	28.81±1.18	33.41±1.14	27.55±1.10	29.42±1.08

Table S5. Compressive strength (*R*, MPa) for the tested dental fillings obtained from photo-cured compositions containing DF1 filler.

Dye	*R* [MPa]			
	PhTAA	NAA	PhAA	EDMAB
Ph1	1.75	1.90	1.20	1.80
Ph2	1.80	1.75	1.40	1.90
Ph3	1.75	1.80	1.25	1.75
Ph4	1.87	1.90	1.55	1.78
CQ	1.50	1.60	1.10	1.70
